# Tumor-Intrinsic PD-L1 Exerts an Oncogenic Function through the Activation of the Wnt/β-Catenin Pathway in Human Non-Small Cell Lung Cancer

**DOI:** 10.3390/ijms231911031

**Published:** 2022-09-20

**Authors:** Yunxia Ma, Rumyana Marinkova, Miljana Nenkov, Lai Jin, Otmar Huber, Jürgen Sonnemann, Natália Peca, Nikolaus Gaßler, Yuan Chen

**Affiliations:** 1 Section Pathology of the Institute of Forensic Medicine, Jena University Hospital, Friedrich Schiller University Jena, Am Klinikum 1, 07747 Jena, Germany; 2 Department of Hematology, Zhejiang Provincial People’s Hospital, Hangzhou 310014, China; 3 Institute of Biochemistry II, Jena University Hospital, Friedrich Schiller University Jena, Nonnenplan 2, 07743 Jena, Germany; 4 Department of Pediatric Hematology and Oncology, Children’s Clinic, Jena University Hospital, Friedrich Schiller University Jena, Am Klinikum 1, 07747 Jena, Germany

**Keywords:** NSCLC, PD-L1, Wnt/β-catenin pathway, exosomal miRNA

## Abstract

Programmed death ligand 1 (PD-L1) strongly inhibits T cell activation, thereby aiding tumors in escaping the immune response. PD-L1 inhibitors have proven to be effective in the treatment of different types of cancer, including non-small cell lung cancer (NSCLC). Yet, the knowledge regarding the biological function of tumor-intrinsic PD-L1 in lung cancer remains obscure. In our study, we set the goal of determining the function of PD-L1 using overexpression and knockdown strategies. PD-L1 silencing resulted in decreased migratory and invasive ability of tumor cells, together with attenuated colony-forming capacity. Ectopic expression of PD-L1 showed the opposite effects, along with increased activities of MAPK and Wnt/β-catenin pathways, and the upregulation of Wnt/β-catenin target genes. Additionally, overexpression of PD-L1 was associated with dysregulated cellular and exosomal miRNAs involved in tumor progression and metastasis. In primary lung tumors, immunohistochemistry revealed that both PD1 and PD-L1 were highly expressed in squamous cell carcinoma (SCC) compared to adenocarcinoma (*p* = 0.045 and *p* = 0.036, respectively). In SCC, PD1 expression was significantly associated with tumor grading (*p* = 0.016). Taken together, our data suggest that PD-L1 may exert an oncogenic function in NSCLC through activating Wnt/β-catenin signaling, and may act as a potential diagnostic marker for lung SCC.

## 1. Introduction

Lung cancer is among the most frequently diagnosed types of cancer, and it remains the leading cause of cancer-related death in both men and women across the globe, contributing to about 1.8 million deaths in 2020 [1].

Histologically, lung cancer is divided into two main types: small cell lung cancer (SCLC) and non-small cell lung cancer (NSCLCs), which include adenocarcinoma (ADC), squamous cell carcinoma (SCC), and large cell lung cancer (LCLC), together accounting for about 85% of all lung cancers. Lung cancer is usually detected in advanced stages of the disease, with limited treatment options and an unfavorable clinical outcome. At the time of diagnosis, most patients already exhibit local and/or distant metastasis [2]. The application of biomarker and low-dose computed tomography screening for high-risk individuals, and refinements in treatment, have led to progress in lung cancer management over the past 10 years. However, the 5-year survival rate continues to be low, at around 25% in NSCLC and 7% in SCLC, for all stages combined after diagnosis (https://www.cancer.org/cancer/lung-cancer/detection-diagnosis-staging/survival-rates.html, accessed data 12 September 2022). Thus, a comprehensive understanding of tumor biology is urgently needed in order to achieve an improvement in lung cancer diagnosis and therapy.

Based on intensive research in oncology, the role of the PD1/PD-L1 axis in maintaining a balance between immune tolerance and immunopathology has emerged in recent years. PD1, an immune checkpoint molecule, is expressed constitutively on T and B lymphocytes, natural killer cells, and dendritic cells [3]. In addition, PD1 expression has also been found in tumor cells [4]. PD-L1 (known as B7-H1 or CD274), acts as a main ligand of the PD1 receptor. Structural analysis has revealed that PD-L1 is a type I transmembrane protein with two extracellular domains (IgV- and IgC-like) and a short cytoplasmic tail involved in the transduction of signals [5]. PD-L1 expression can be induced by a variety of factors, including oncogenic mutations and IFNγ via Jak/STAT or PI3K/Akt signaling [5]. Recent studies indicate that PD-L1 is expressed in 30–60% of NSCLC cases, although these numbers vary across publications [6,7].

A growing body of evidence shows that lung cancer cells exploit several mechanisms for immune evasion, mainly by the production of immunosuppressive cytokines and the expression of T cell-activation-inhibiting molecules, most notably CTLA-4 (cytotoxic T lymphocyte-associated 4) and PD1/PD-L1 [8]. The anti-PD1/PD-L1 treatment blocks the interaction of PD1 and its ligands, restores the vitality of the T cells, and thereby exerts the anti-tumor immune effect [9]. Clinically, immunotherapy with monoclonal antibodies against PD1/PD-L1 has improved clinical outcomes of metastatic NSCLC patients whose tumor cells display certain biological characteristics, such as PD-L1 expression, high tumor mutational burden, or mismatch repair deficiency/microsatellite instability [10,11,12].

The majority of studies on PD-L1 have thus far focused on its role in immunity and immunotherapy, while less attention has been paid to its tumor-intrinsic function in human cancer. In the present study, we used “gain-of-function” and “loss-of-function” strategies, as well as exosomal microRNA analysis, with the aim of shedding more light on the functional role of PD-L1 in NSCLC.

## 2. Results

### 2.1. PD1 and PD-L1 Expression in Lung Cancer

**Figure 1:**
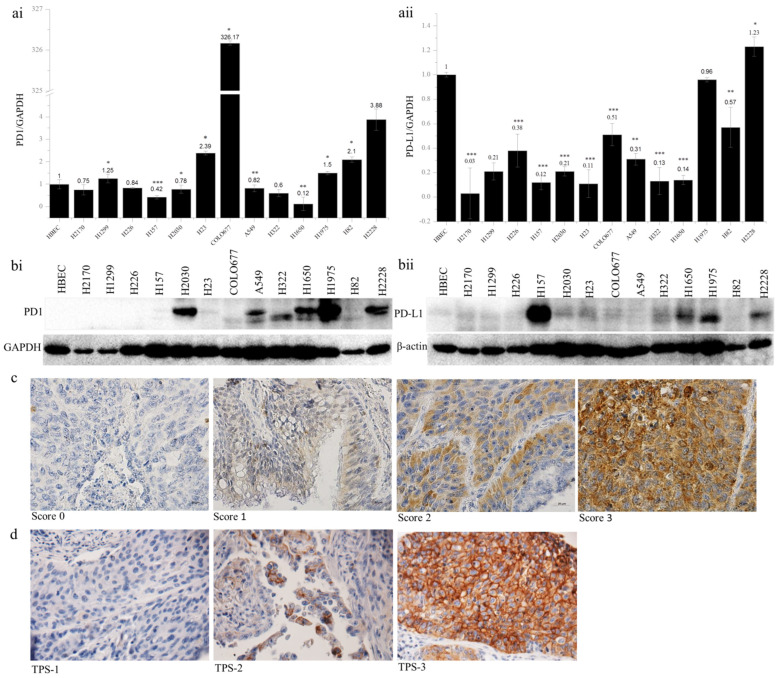
PD1 and PD-L1 expression in human lung cancer cell lines and primary lung tumors. *PD1* (**ai**) and *PD-L1* (**aii**) mRNA expression analyzed by quantitative real-time RT-PCR. The *PD1* and *PD-L1* mRNA expression was normalized to the expression level of *GAPDH* as an internal control, which in normal human bronchial epithelial cells (HBEC) was defined as 1.0; * *p* < 0.05, ** *p* < 0.01, *** *p* < 0.001 when analyzed using Student’s *t*-test. PD1 (**bi**) and PD-L1 (**bii**) protein expression analyzed by Western blotting. β-actin was used as a loading control. Representative images from immunohistochemical staining of PD1 (**c**) and PD-L1 (**d**) in primary lung tumor samples. TPS, tumor proportion score. Magnification, 400×.

The expression of PD1 and PD-L1 was analyzed in 13 lung cancer cell lines by quantitative real-time RT-PCR and Western blotting (WB). Compared to the normal bronchial epithelial cells (HBEC), the expression of *PD1* mRNA was upregulated in six lung cancer cell lines, including H1299, H23, COLO677, H1975, H82, and H2228, with the expression level being more than 326-fold higher in COLO677 (Figure 1(ai)). In contrast to *PD1*, the expression of *PD-L1* mRNA was widely downregulated in 12 cell lines, except for H2228 (Figure 1(aii)). At the protein level, PD1 was upregulated in H2030, A549, H322, H1650, H1975, and H2228 (Figure 1(bi)), while PD-L1 was obviously upregulated in H157, H1650, H1975, and H2228, compared to HBEC (Figure 1(bii)). The discordant expression between mRNA and protein levels indicate post-transcriptional regulation of PD1 and PD-L1 in lung cancer cells.

To determine whether *PD1* and *PD-L1* are epigenetically regulated, 11 out of 13 lung cancer cell lines were treated with a DNA methyltransferase inhibitor, 5-aza-2′-deoxycytidine (5-Aza), and a histone deacetylase inhibitor, Trichostatin A (TSA), respectively. After treatment with 5-Aza, the *PD1* expression was significantly increased in eight lung cancer cell lines (H2170, H1299, H226, H157, H23, A549, H1650, and H1975), and *PD-L1* expression was significantly enhanced in nine lung cancer cell lines (H2170, H1299, H226, H157, H2030, COLO677, A549, H1650, and H1975) (Supplementary Figure S1). Similarly, TSA treatment led to the significant upregulation of *PD1* and *PD-L1* in nine and seven lung cancer cell lines, respectively (Supplementary Figure S2).

The results indicate that epigenetic mechanisms may be involved in the dysregulation of *PD1* and *PD-L1* in lung cancer cells. To further clarify the role of DNA methylation, we performed bisulfite sequencing. However, neither *PD1* nor *PD-L1* was found to be methylated in the promoter region in lung cancer cell lines.

The protein expression of PD1 and PD-L1 was analyzed by immunohistochemistry on TMAs. In total, 24 out of 131 samples (18.3%) exhibited PD1 expression (Table 1). The expression levels did not differ from gender or TNM stages, but they were significantly associated with tumor subtypes and differentiation status. PD1 expression was significantly higher in SCC than in ADC (*p* = 0.045), and in SCC, higher expression of PD1 was significantly related to lower tumor grading (*p* = 0.016, Table 2). In view of the PD-L1 expression (Table 3), 30 (two ADC and 28 SCC) out of 119 samples (ADC + SCC) showed positive staining in the membranes of tumor cells. This difference regarding PD-L1-positive staining between ADC and SCC reached statistical significance (*p* = 0.036). Additionally, the positive staining of PD-L1 was correlated with tumor size; however, this was not statistically significant (*p* =0.055). Furthermore, PD1 and PD-L1 expression was significantly correlated (*p* = 0.000, Table 4). Representative images of PD1 and PD-L1 staining are shown in Figure 1c,d. Survival analysis using Kaplan–Meier curves indicated that neither PD1 nor PD-L1 expression was related to the clinical outcome of NSCLC patients (Supplementary Figure S3).

### 2.2. PD-L1 Overexpression Influences Colony Formation, Cell Cycle Proliferation, Migration, and Invasion

**Figure 2:**
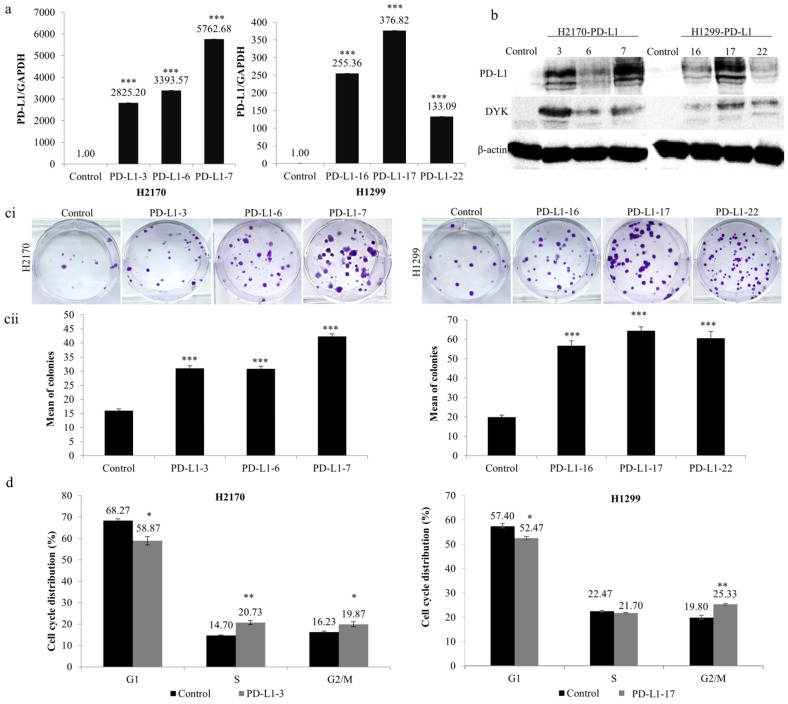
Influence of PD-L1 on colony formation and cell cycle progression in NSCLC cells. Overexpression of (**a**) *PD-L1* mRNA and (**b**) PD-L1 protein in H2170 and H1299 cells after stable transfection, determined by real-time RT-PCR and Western blotting (WB), respectively. β-actin was used as a loading control for WB. PD-L1-3 (3), PD-L1-6 (6), and PD-L1-7 (7) are positive clones from H2170; PD-L1-16 (16), PD-L1-17 (17), and PD-L1-22 (22) are positive clones from H1299. Control cells were transfected with an empty vector. (**c**) PD-L1 overexpression increased H2170 and H1299 proliferation, as revealed by colony formation assay. Representative images of colony formation (**ci**; magnification 50 ×) and quantification of the number of colonies (**cii**). (**d**) Cell cycle analysis by flow cytometry showing that ectopic PD-L1 expression resulted in a significantly increased H2170 cell population in S and G2/M phases and increased H1299 cell numbers in the G2/M phase. * *p* < 0.05, ** *p* < 0.01, *** *p* < 0.001 when analyzed using Student’s *t*-test.

To analyze the function of PD-L1 in NSCLC cells, stable transfection was performed using the expression vector of PD-L1 in H2170 (SCC) and H1299 (ADC), which exhibit no endogenous expression of the PD-L1 protein (Figure 1(bii)). After transfection, overexpression of PD-L1 was confirmed for both mRNA (Figure 2a) and protein levels (Figure 2b). We selected three positive clones, respectively, from the SCC and ADC cell lines (PD-L1-3, PD-L1-6, and PD-L1-7 from H2170; PD-L1-16, PD-L1-17, and PD-L1-22 from H1299) for the colony formation, migration, and invasion assays.

As shown in Figure 2c, ectopic expression of PD-L1 led to significantly increased colony-forming ability in both cell lines compared to controls. Cell cycle analysis revealed that the distribution of cells throughout the three main phases (G1, S, and G2/M) had changed significantly. As evident in Figure 2d, ectopic expression of PD-L1 led to noticeably reduced cell numbers in the G1 phase (both H2170 and H1299), whereas it increased the number of cells in the S phase (H2170) and G2/M phase (both H2170 and H1299). The data from the colony formation assay and cell cycle analysis suggest that PD-L1 may enhance cell proliferation in NSCLC cells.

**Figure 3:**
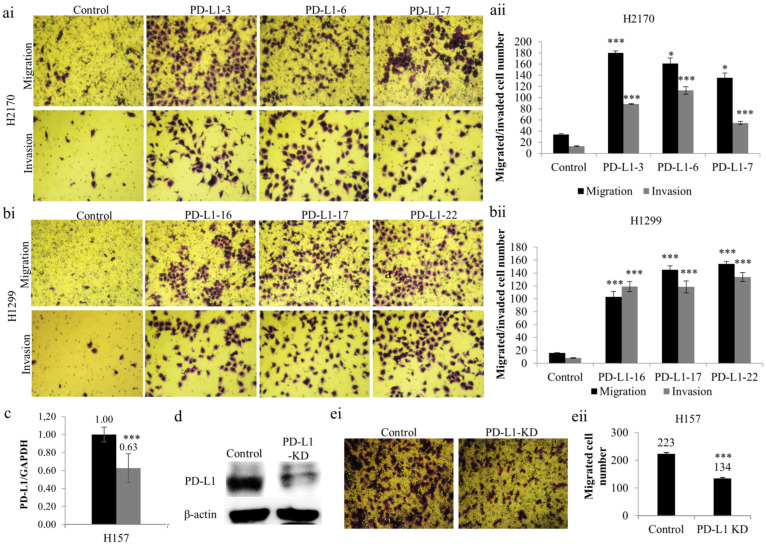
Effects of PD-L1 on NSCLC migration and invasion. Overexpression of PD-L1 enhanced the migratory and invasive ability of H2170 (**a**) and H1299 (**b**) cells. Representative images of the migration and invasion assays (**ai**,**bi**); magnification 50 ×); quantification of the number of migrated and invaded cells of H2170 (**aii)** and H1299 **(bii**). Reduced *PD-L1* mRNA expression (**c**) and protein expression (**d**) in H157 cells after siRNA knockdown (KD). β-actin was used as a loading control for Western blotting (WB). (**e**) Knockdown of PD-L1 led to decreased migratory ability, revealed by migration assay. Representative images of the migration assay (**ei,** magnification 50×); quantification of the number of invaded cells (**eii**). * *p* < 0.05, *** *p* < 0.001 when analyzed using Student’s *t*-test.

Additionally, a significantly greater number of PD-L1-overexpressing cells revealed an augmented migratory ability (Figure 3a,b) in comparison to mock cells. Similarly, the number of cells that migrated through the Matrigel (Figure 3a,b) was significantly increased in H2170 and H1299 cells stably transfected with PD-L1, which reveals an enhanced tumor invasive property induced by PD-L1 overexpression. Thus, the results collectively outline an oncogenic role of PD-L1 in NSCLC cells.

### 2.3. Knockdown of PD-L1 by siRNA Decreases the Migration and Invasion Ability of NSCLC Cells

To further confirm the role of PD-L1 as an oncogene, we performed “loss-of-function” analysis to knock down PD-L1 using siRNA in H157 cells endogenously expressing the PD-L1 protein. PD-L1 siRNA transfection led to considerably decreased gene expression at both mRNA (Figure 3c) and protein (Figure 3d) levels, indicating successful gene silencing by siRNA knockdown. The reduction in PD-L1 expression at the protein level is more obvious than that at the mRNA level, reflecting the post-transcriptional regulation of PD-L1.

As mentioned above, migration assays were performed. It was found that PD-L1 gene silencing resulted in reduced migratory ability in H157 compared to mock cells (Figure 3(ei,eii)). The findings confirm the oncogenic activity of PD-L1 in NSCLC cells, particularly concerning tumor cell migration, an initial step in metastasis.

### 2.4. PD-L1 affects MAPK and Wnt/β-Catenin Tumorigenic Pathways and Cell-Cycle-Associated Genes

To explore the signaling pathways involved in the PD-L1-mediated enhancement of tumor cell proliferation, migration, and invasion, we analyzed the influence of PD-L1 overexpression on tumorigenic pathways, including mitogen-activated protein kinase (MAPK), Akt/mTOR, and Wnt/β-catenin pathways.

**Figure 4:**
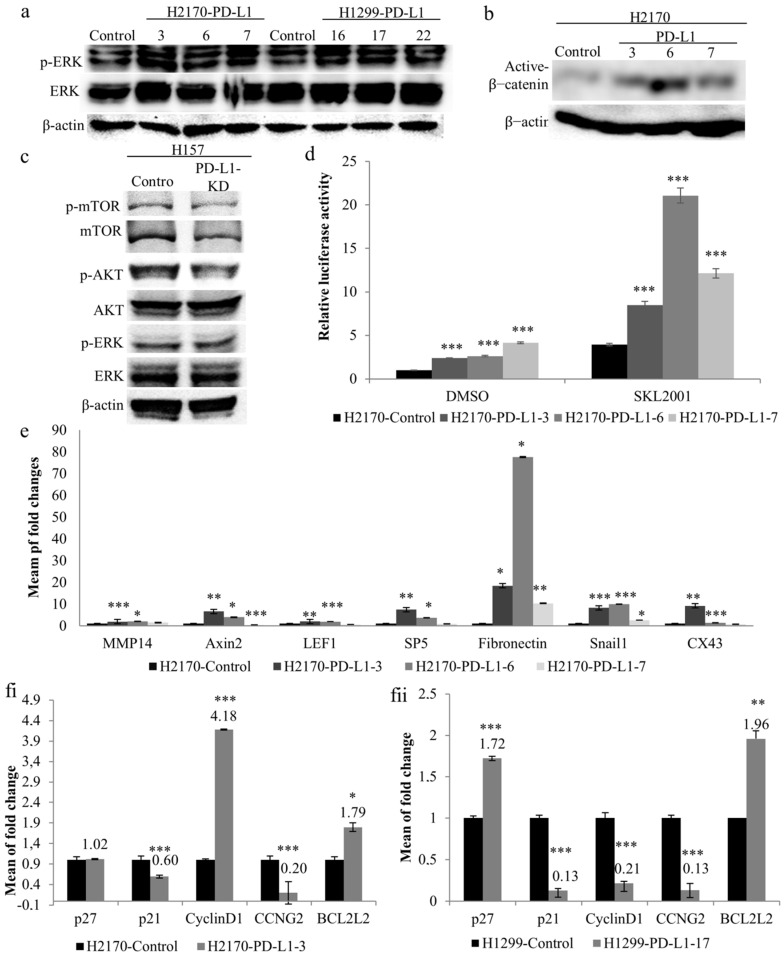
Impact of PD-L1 on oncogenic signaling pathways and cell cycle regulatory genes. (**a**) Ectopic PD-L1 expression led to increased phosphorylated levels of ERK in both H2170 and H1299 cells, as revealed by Western blotting (WB). β-actin was used as a loading control. PD-L1-positive transfectants 3, 6, and 7 were from H2170; PD-L1-positive transfectants 16, 17, and 22 were from H1299. (**b**) H2170 PD-L1-positive transfectants 3, 6, and 7 showed higher protein expression levels of active β-catenin compared to control cells. (**c**) Knockdown of PD-L1 in H157 cells resulted in decreased phosphorylated Akt. (**d**) Significantly enhanced Wnt/β-catenin activity in PD-L1-overexpressing H2170 cells, as determined by TCF/LEF reporter gene assays. The activity of mock-transfected H2170 cells treated with DMSO was set to 1. SKL2001 is an agonist of the Wnt/β-catenin pathway. (**e**) Increased expression levels of Wnt/β-catenin pathway downstream target genes in H2170 after PD-L1 transfection, revealed by real-time RT-PCR analysis. Alteration of cell cycle regulatory gene expression in PD-L1-transfected H2170 (**fi**) and H1299 (**fii**), compared to control cells, using real-time RT-PCR analysis. * *p* < 0.05, ** *p* < 0.01, *** *p* < 0.001 when analyzed using Student’s *t*-test.

As illustrated in Figure 4a, ectopic expression of PD-L1 led to increased phosphorylated levels of ERK1/2 in the three positive transfectants of H2170 and H1299, respectively, compared to mock cells, indicating a regulatory role of PD-L1 in the MAPK pathway. Additionally, active β-catenin expression levels were found to be upregulated by PD-L1 in all three positive clones from H2170 (Figure 4b), but not from H1299. Knockdown of PD-L1 resulted in a reduced level of phosphorylated Akt in H157 cells, while changes in phosphorylated mTOR and ERK were not obvious, compared to control cells (Figure 4c, Supplementary Figure S4).

To further elucidate the effects of PD-L1 on Wnt/β-catenin signaling, we performed luciferase reporter assays using a Dual-Luciferase Reporter Gene System. Significantly enhanced luciferase activity was found in the three PD-L1-positive clones from H2170 (Figure 4d) and one PD-L1-positive clone from H1299 (Supplementary Figure S5), compared to control cells. Upon treatment with SKL2001, an agonist of the Wnt/β-catenin pathway, the luciferase activity increased dramatically in both PD-L1-transfected cells and mock cells; however, compared to mock cells, the PD-L1-transfected cells still exhibited significantly higher activity. We also observed that positive clone 6 showed the highest luciferase activity after SKL2001 stimulation, in line with it exhibiting the highest expression level of active β-catenin.

We then analyzed the mRNA expression of downstream target genes of the Wnt/β-catenin pathway in PD-L1-transfected cells. In the PD-L1-transfected H2170 cells, significantly increased expression of the Wnt/β-catenin target genes, including *Axin2*, *Snail1*, and *fibronectin*, was found in all the three positive clones, and at least two positive clones showed significant upregulation of *MMP14*, *LEF1*, *SP5*, and *CX43*, compared to mock cells (Figure 4e). In the PD-L1-transfected H1299 cells, *MMP14*, *Axin2*, and *fibronectin* were upregulated (Supplementary Figure S6). The discrepancy between H2170 and H1299 cells regarding the expression of active β-catenin and the Wnt/β-catenin target gene upon PD-L1 overexpression may reflect cell-type specificity.

To determine the molecular alterations during cell cycle progression, we analyzed the mRNA expression of cell-cycle-associated genes. As shown in Figure 4(fi,fii), Cell cycle regulators and apoptosis-related genes, such as cyclin D1 and BCL2L1, were upregulated, while *p21* and *CCNG2* were downregulated in PD-L1-overexpressing H2170 cells, compared to control cells. In the PD-L1-transfected H1299 cells, *p27* and *BCL2L1* were overexpressed, while *p21*, *cyclin D1*, and *CCNG2* expression was reduced. Again, this difference may be related to cell-type-dependent regulation.

Taken together, our data indicate that certain oncogenic pathways are involved in the PD-L1-mediated tumor-promoting effects of NSCLC. Among them, the Wnt/β-catenin signaling pathway may be particularly important for facilitating PD-L1-mediated NSCLC cell proliferation, migration, and invasion.

### 2.5. PD-L1 Regulates Cellular and Exosomal miRNAs Involved in Tumor Migration, Invasion, and Metastasis

Recent studies have demonstrated that both cellular and exosomal microRNAs regulate the expression of genes that play essential roles in cancer cell migration, invasion, and metastasis [13,14].

**Figure 5:**
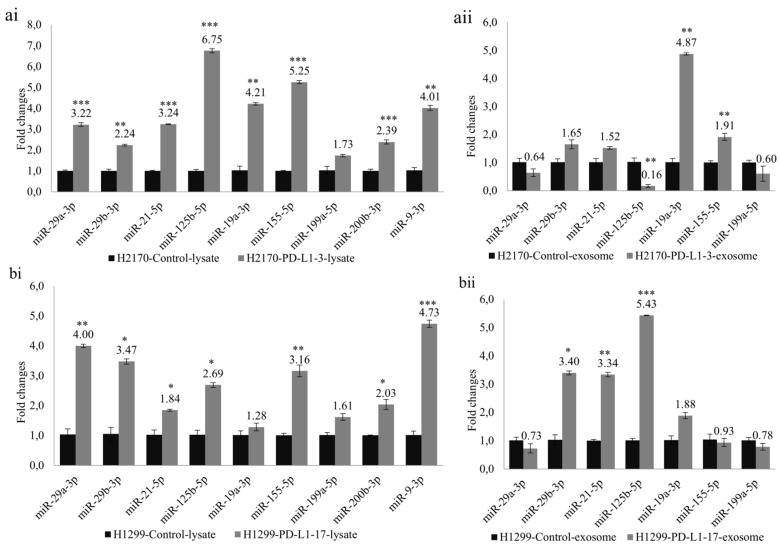
Analysis of cellular miRNAs and exosomal miRNAs revealed by real-time RT-PCR in PD-L1 transfected cells. Ectopic expression of PD-L1 significantly changed the expression levels of miRNAs participating in tumor cell progression and metastasis. Altered expression levels of cellular miRNAs in H2170 (**ai**) and H1299 (**bi**). Altered expression levels of exosomal miRNAs in H2170 (**aii**) and H1299 (**bii**). For cellular miRNA analysis, U6 was used as an internal control; for exosomal miRNA analysis, spike-in protein Sp6 was used as an internal control. * *p* < 0.05, ** *p* < 0.01, *** *p* < 0.001.

To investigate whether PD-L1-mediated tumor cell growth is accompanied by dysregulated miRNAs, we selected nine miRNAs (miR-29a-3p, miR-29b-3p, miR-21-5p, miR-125b-5p, miR-19a-3p, miR-155-5p, miR-199a-5p, miR-200b-3p, and miR-9-3p) that participate in tumor invasion and metastasis. Ectopic PD-L1 expression led to markedly elevated expression levels of cellular miR-29a-3p, miR-29b-3p, miR-21-5p, miR-125b-5p, miR-19a-3p, miR-155-5p, miR-200b-3p, and miR-9-3p in H2170 transfectants (Figure 5(ai)). Similarly, in H1299 cells transfected with PD-L1, the cellular levels of miR-29a-3p, miR-29b-3p, miR-21-5p, miR-125b-5p, miR-155-5p, miR-200b-3p, and miR-9-3p were notably upregulated (Figure 5(bi)).

Before expression analysis of exosomal miRNAs, exosomes were characterized by Western blotting using antibodies against CD9 and CD63, two widely recognized markers for exosomes (Supplementary Figure S7). For the exosomal miRNA analysis, we excluded miR-200b-3p and miR-9-3p due to their very low expression levels in the exosomes. In H2170 cells, overexpression of PD-L1 resulted in significantly enhanced expression levels of exosomal miR-19a-3p and miR-155-5p, but a decreased level of miR-125b-5p, compared to mock control cells (Figure 5(aii)). In PD-L1-overexpressing H1299 cells, exosomal miR-29b-3p, miR-21-5p, and miR-125b-5p were upregulated (Figure 5(bii)). The expression pattern of exosomal miRNAs differed from that of cellular miRNAs.

## 3. Discussion

The application of immune checkpoint inhibitors is associated with improved overall survival, a mild safety profile, and superiority over conventional therapies for metastatic melanoma, colorectal carcinoma, non-small cell lung carcinoma (NSCLC), bladder carcinoma, renal cell carcinoma, and Hodgkin lymphoma [15,16]. Additional to the role of PD-L1 in immune regulation, tumor-intrinsic PD-L1 exerts non-immunological functions in regulating mesenchymal transition, glucose and lipid metabolism, stemness, and autophagy [17]. In this study, we explored the function of tumor-intrinsic PD-L1 in human NSCLC.

The expression analysis revealed that *PD-L1* mRNA was widely downregulated in a panel of lung cancer cell lines, except H2228, compared to normal HBEC. At the protein level, the SCC cell line H157 showed the highest expression of *PD-L1*. In line with the observation that tumor-intrinsic PD1 is widespread in certain cancer types, including melanoma, hepatic cell carcinoma, and NSCLC [18], we found that the protein expression levels of PD1 were significantly increased in six lung cancer cell lines. The different expression patterns of PD1/PD-L1 imply their different functions in NSCLC. Increasing evidence shows that NSCLC in patients with PD-L1 overexpression (TPS > 50%) is significantly related to tobacco smoking, and these patients experience better responses to immunotherapy with PD-L1 inhibitors [19,20]. However, we could not analyze the relationship between PD-L1 expression and smoking, since the smoking history of the NSCLC patients included in the study was not available.

Methylation of the PD-L1 gene promoter represents a potential diagnostic and prognostic marker in gastrointestinal carcinoma [21,22]. In NSCLC, PD-L1 expression was found to be regulated by both DNA methylation and NF-kB during epithelial–mesenchymal transitions [23]. In head and neck cancer, PD1 DNA methylation is associated with human papillomavirus prognosis, mutational load, and immune infiltrates [24]. These findings imply an epigenetic regulation of PD1/PD-L1 in cancer cells. In our study, despite the upregulation of PD1/PD-L1 upon treatment with a DNA methyltransferase inhibitor and a histone deacetylase inhibitor, bisulfite sequencing did not reveal any promoter methylation. This might be explained by the fact that neither cis-regulatory element in the PD1 nor PD-L1 promoter is methylated, but promoters of their upstream regulatory genes are methylated, leading to an increased gene expression after demethylation. Anyhow, to address the epigenetic mechanism accurately, more CpG islands of PD1/PD-L1 DNA should be included in future investigations.

A compelling body of research indicates an immune-independent tumor cell proliferation and oncogenic signals triggered by intrinsic PD-L1 in melanoma, ovarian cancer, and head and neck cancer [25,26,27]. In line with these findings, our data show that overexpression of PD-L1 was associated with enhanced colony formation, migration/invasion, and cell cycle progression, while knockdown of PD-L1 led to opposite behaviors of the NSCLC cells, suggesting that PD-L1 may act as an oncogene to regulate immune-independent tumor growth in NSCLC.

In the exploration of PD-L1-dependent signaling pathways, we found that several oncogenic pathways were markedly affected by PD-L1. Ectopic expression of PD-L1 caused increased phosphorylation of ERK, a key player of the MAPK pathway, and activation of Wnt/β-catenin pathway, evident with an enhanced level of active β-catenin, increased Wnt/β-catenin activity, and upregulation of downstream target genes. The PD-L1 gene silencing, however, resulted in a decreased AKT activity. In breast cancer cells, overexpression of PD-L1 was reported to contribute to chemoresistance and stemness-like properties via activating PI3K/AKT and MAPK/ERK pathways [28]. In line with our findings, the regulatory role of PD-L1 on Wnt/β-catenin signaling was depicted in colorectal carcinoma (CRC) and NSCLC. In CRC, PD-L1 interacting with Frizzled 6, activated β-catenin to promote cancer stem cell expansion [29]. In NSCLC, PD-L1 was able to stabilize β-catenin through the activation of PI3K/AKT and MAPK/ERK signaling pathways [30]. Wnt/β-catenin and MAPK signaling can be allies or enemies in different battlefields [31]. The crosstalk between Wnt/β-catenin and MAPK pathway or other oncogenic pathways may affect their target gene expression. On the other hand, activation of β-catenin was found to induce PD-L1 transcription and promote tumor cell immune evasion [32]. These data imply a bidirectional positive feedback loop between PD-L1 and β-catenin, and provide hints for therapeutic implications. Indeed, blocking Wnt/β-catenin signaling amplified anti-PD-1 therapeutic efficacy by inhibiting tumor growth, migration, and promoting immune infiltration in glioblastomas [33].

A vast number of studies demonstrate that microRNAs may regulate PD-L1 in two different ways: directly targeting the 3′-UTR of PD-L1 mRNA or indirectly via signaling molecules [34]. Yet, knowledge on the regulatory role of PD-L1 on miRNAs appears to be sparse. In our study, we analyzed the expression of several miRNAs involved in lung cancer migration, invasion, and metastasis in the context of PD-L1 overexpression. MiR-29b attenuates NSCLC metastasis by targeting matrix metalloproteinase 2 and PTEN [35]. MiR-21 promotes NSCLC cell progression by downregulating SOCS1, SOCS6, and PTEN, associated with poor prognosis of primary NSCLC [36]. MiR-125b promotes tumor metastasis through targeting tumor protein 53-induced nuclear protein 1 in NSCLC [37]. Overexpression of miR-19a/b is related to the poor prognosis and metastasis of pulmonary cancer cells [38]. MiR-155-5p suppresses the migration and invasion of lung adenocarcinoma cells by targeting Smad2 [39]. MiR-199a-5p suppresses NSCLC progression [40], while miR-200b-3p and miR-9 promote NSCLC metastasis by targeting ABCA1 and TGFBR2, respectively [41,42]. In the study, most of the selected miRNAs were upregulated in the PD-L1-transfected cells. By comparison with the cellular miRNA expression between H2170 and H1299 cells, we found that the miRNA expression patterns are similar but not exactly identical, which might be related to the context of cell-type specificity. In addition to cellular miRNAs, exosomal miRNAs were included in our analyses, since they are one of the important molecular cargos transmitted by exosomes to facilitate cell-cell communication. It was found that the expression patterns between lysate miRNAs and exosomal miRNAs were not the same, however, they shared some similarities. For example, in H2170 cells transfected with PD-L1, a remarkable upregulation of miR-19a-39p and miR-155-5p was found in both cell lysates and exosomes, but other miRNAs showed diverse expression levels between lysates and exosomes, compared to the mock-transfected control cells. It is not surprising to see this discrepancy. Exosome motifs and the 3′-end sequences of miRNA determine the process of miRNA loading into exosomes, which may result in altered miRNA enrichment within exosomes, compared to their original cells [43]. This diversity has also been observed in clinical samples derived from laryngeal squamous cell carcinoma, in which microRNA expression profiles differed significantly from parental cells to exosomes [44]. Overall, in the translational context, these tumor-associated miRNAs could be potential therapeutic targets for NSCLC.

Clinically, PD-L1 expression has been considered as a predictive biomarker to stratify NSCLC patients for immunotherapy, albeit with limitations and challenges [45,46]. In line with previous reports [47,48], our data show that PD-L1 might be a potential diagnostic marker for lung SCC, since SCC samples exhibited significantly more positive staining of PD-L1, compared to lung ADC. The correlation between PD1 and PD-L1 in vivo turned out to be significantly positive (Table 4), in line with the in vitro findings that PD-L1 overexpression led to significantly increased levels of the PD1 protein (Supplementary Figure S8). For example, a blockade of tumor-expressed PD1 promotes lung cancer growth, while inhibits melanoma and hepatocellular cancer cell growth [18]. The influence of the multifaceted functions of PD1 beyond immune checkpoint signaling on the anti-PD1/PD-L1 therapeutic efficiency is worth to be studied further.

In summary, PD-L1 exerts a tumor-promoting function in NSCLC through the activation of oncogenic pathways. The PD-L1-induced changes in intracellular signaling beyond immunity may define new mechanisms through which the PD1/PD-L1 axis facilitates tumor cell progression and development and indicate new therapeutic strategies for patients with lung cancer.

## 4. Materials and Methods

### 4.1. Cell Culture

Thirteen NSCLC cell lines, including eight ADC (H1299, H2030, H23, A549, H322, H1650, H1975, and H2228) and three SCC (H2170, H226, and H157) lines, together with two SCLC cell lines (COLO677 and H82), were purchased from the American Type Culture Collection (ATCC, Rockville, MD, USA) and the German Collection of Microorganisms and Cell Culture (DSMZ, Braunschweig, Germany). The cell lines were grown in RPMI 1640 medium supplemented with 10% (*v*/*v*) fetal bovine serum (FBS) (Thermo Fisher, Hamburg, Germany). For the transfected cells, 400 ng/µL of Geneticin (G418) (Santa Cruz Biotechnology, Dallas, TX, USA) was added into the cell culture medium. Normal human bronchial epithelial cells (HBEC) were obtained from Lonza (Cologne, Germany) and grown in BEG media (Cologne, Germany). Cells were maintained in a humidified incubator with 5% CO_2_ at 37 °C.

For drug treatment, 11 lung cancer cell lines were seeded in six-well plates. When the cells reached 50% confluence, they were treated with a DNA methyltransferase inhibitor, 5-aza-2′-deoxycytidine (5-Aza) and a histone deacetylase inhibitor, Trichostatin A (TSA) (Santa Cruz Biotechnology, Dallas, TX, USA), respectively, as previously described [49].

### 4.2. RNA Extraction and Real-Time RT-PCR

RNA extraction from cell lines and exosomes was performed using Trizol reagent (Ambion, Carlsbad CA, USA) and reversely transcribed to cDNA using a QuantiTect^®^ Reverse Transcription Kit (Qiagen, Hilden, Germany) and a miRCURY lNA RT Kit (Qiagen, Hilden, Germany), according to the manufacturer’s protocols.

Real-time RT-PCR was conducted on the Rotor-Gene Q (Qiagen, Hilden, Germany) using a FastStart Universal SYBR Green Master (Merck, Munich, Germany) for gene expression analysis and a miRCURY LNA SYBR Green PCR Kit for miRNA analysis, following the manufacturer’s guidance. Glyceraldehyde-3-phosphate dehydrogenase (*GAPDH*) or U6 was used as an internal control for gene expression (mRNA) or miRNA expression analysis. For exosomal miRNA analysis, the Sp6 spike-in protein (Qiagen, Hilden, Germany) was used as an internal control. Real-time RT-PCR results were quantified using the 2-ΔΔCT method [50]. The data presented were derived from three independent experiments. The primer sequences are available in Supplementary Table S1.

### 4.3. Genomic DNA Isolation, Bisulfite Treatment, and Bisulfite Sequencing

Genomic DNA isolation, bisulfite treatment, and bisulfite sequencing (BS) were conducted as described previously [51]. The primers used for bisulfite sequencing are included in Supplementary Table S1.

### 4.4. Protein Extraction and Western Blot Analysis

Proteins from the cells were extracted using RIPA buffer (Merck, Munich, Germany). The concentrations of the proteins were determined using a BCATM Protein Assay Kit (Thermo Scientific, Rockford, IL, USA). Western blot analysis was carried out as described previously [52]. In total, 30 µg of proteins were electrophoresed on 8%, 12%, or 15% SDS-PAGE gels according to the size of the proteins, and transferred on nitrocellulose membranes. Antibodies applied for the Western blot analysis are listed in Supplementary Table S2. An enhanced chemiluminescence detection system (Santa Cruz Biotechnology, Dallas, TX, USA) was used to detect signals according to the manufacturer’s recommendations. β-actin served as a loading control.

### 4.5. Immunohistochemistry on Tissue Microarrays (TMAs)

TMAs containing 131 primary lung tumor samples obtained from Hospital Bad Berka from 1999 to 2002 were constructed using a manual tissue arrayer (Beecher Instruments, Woodland, WI, USA) as previously described [53]. Neither adjuvant radiotherapy nor chemotherapy was performed before surgical operations. This study was approved by the local ethical committee of University Hospital Jena (Nr.: 3815-07/13).

Protein expression of PD1 and PD-L1 was analyzed by immunohistochemistry (IHC) on the lung tumor TMA. Briefly, after deparaffinization with xylene and gradual hydration, antigen retrieval was carried out by treatment of the tissue sections in a pressure cooker for 6 min. The working concentrations of the antibodies are presented in Supplementary Table S2. Signal detection was conducted according to the manufacturer’s protocol (LSABTM 2-kits, DAKO, Hamburg, Germany). For the expression of PD1, immunohistochemistry was scored semiquantitatively, with less than 5 % positively stained cells classed as negative staining (score 0), 5–25% positively stained cells as weak staining (score 1), 26–50% positively stained cells as moderate staining (score 2), and more than 50 % positively stained cells as strong staining (score 3). For the statistical analysis, scores 0 and 1 together were considered negative, while scores 2 and 3 were grouped together as positive. Regarding the expression of PD-L1, a tumor proportion score (TPS) less than 1% (TPS1) was considered negative expression, TPS2 (1–49% positive staining) was considered low or moderate expression, and TPS3 (≥50% positive staining) was viewed as high expression. For the statistical evaluation, TPS1 was defined as negative, while TPS2 and TPS3 were grouped together as positive.

### 4.6. Stable Transfection

An expression vector containing the full-length cDNA of the human PD-L1 gene was obtained from GenScript (Piscataway, NJ, USA). It was transfected into a lung SCC cell line H2170 and an ADC cell line H1299 using lipofectamine^®^ 2000 DNA Transfection Reagent (Thermo Fisher, Hamburg, Germany). Both cell lines exhibit no endogenous expression of PD-L1. An empty vector pcDNA3.1 (GenScript, Piscataway, NJ, USA) was used as a mock control. Stable transfection was carried out by adding G418 (400 µg/mL) (Santa Cruz, Santa Cruz, Dallas, TX, USA) for the selection of positive clones, as previously described [54].

### 4.7. Colony Formation Assay, Cell Migration and Invasion Assays

In order to determine whether PD-L1 plays a role in cell proliferative ability, PD-L1-transfected cells and mock cells from H2170 and H1299 were subjected to a colony formation assay. Cells were seeded in six-well plates at a density of 3000 cells/well and incubated for 10–11 days in a 37 °C incubator with 5% CO_2_ to allow colony formation, followed by fixation and staining with 0.5% crystal violet. The number of colonies was counted under microscope. A cluster containing around 50 cells represents one colony.

To assess the migratory potential of the transfected cells, 3 × 104 cells/well) were resuspended in 500 μL FBS-free cell culture medium and seeded in the upper transwell chamber (8 μm pore size, Corning^®^, Discovery Labware, Corning, NY, USA). Medium that contained 20% (*v*/*v*) FBS was placed in the lower chamber and acted as a chemoattractant. The upper chambers were then placed in a 24-well culture dish with 700 µL of cell culture medium, and the cells were incubated at 37 °C for 24 h. Afterwards, the non-migratory cells were removed using a cotton swab. The migratory cells were fixed, stained with crystal violet at 37 °C for 10 min, and counted.

In parallel to the migration assay, an invasion assay using Corning^®^ BioCoat™ Matrigel^®^ Invasion Chambers (Discovery Labware, Inc.) was performed. Cells (3 × 104 cells/well) were cultured in FBS-free medium for 24 h and seeded in Matrigel-coated transwell chambers (BD Biosciences, Franklin Lakes, NJ, USA). After incubation at 37 °C for 24 h, uninvaded cells were removed, and invaded cells were stained and counted.

For cell cycle analysis, cells (1 × 105 cells/well) were seeded in six-well plates and incubated at 37 °C for 72 h. Cells were centrifuged at 1100 rpm for 5 min at room temperature and fixed with pre-cold 70% ethanol. Following fixation, cells were transferred into 5 mL polystyrene round-bottom test tubes (Discovery Labware, Inc.) and centrifuged at 1800 rpm at 4 °C for 5 min. After washing, the cell pellets were resuspended in PBS containing 50 μg/mL RNase A, 1% (*w/v*) glucose, and 50 μg/mL propidium iodide, and incubated at 4 °C for 45 min in darkness. Cell cycle analysis was carried out using a BD FACSCanto™ II flow cytometer (BD Biosciences, Heidelberg, Germany). Data were gated to exclude debris, and the different cell cycle phases were quantified using FACSDiva (BD Biosciences) software.

All the assays were performed in triplicate in two independent experiments.

### 4.8. RNA Interference (siRNA)

H157 cells exhibiting endogenous expression of PD-L1 were selected for the “loss-of-function” analysis by siRNA knockdown. The cells were seeded in a six-well plate (3.5 × 105 cells/well) and incubated for 24 h. After that, the cells were transiently transfected with PD-L1 siRNA (sc-39699, Santa Cruz Biotechnology, Dallas, TX, USA) or control siRNA (sc-37007, Santa Cruz Biotechnology, Dallas, TX, USA) using Lipofectamine 2000 (Thermo Fisher, Hamburg, Germany). After incubation for 48 h, cells were harvested for RNA and protein extraction.

### 4.9. TCF/LEF Activity—Luciferase Reporter Assay

TCF/LEF luciferase reporter vector, known as a Wnt pathway responsive reporter, was used to investigate Wnt signaling. Briefly, H2170 and H1299 cells, including both the mock and PDL1 transfectants, were transiently co-transfected with pGL4.49[luc2P/TCF-LEF-RE/Hygro] (Promega; E6921) and pGL4.74 [hRluc/TK] (Promega; E4611) (ratio 10:1) using Lipofectamine^®^2000 transfection reagent (Invitrogen) following the manufacturer’s instructions. Subsequently, 24 h after transfection, cells were treated with the Wnt agonist SKL2001 (CAYMAN CHEMICAL COMPANY, catalog number 26334) or DMSO control for 24 h. Luciferase activity was assessed using the Dual-Luciferase Reporter Assay System (Promega; E1910), and luminescence was measured using the Infinite^®^ 200 Pro (Tecan) reader. Firefly luciferase activity was normalized to *Renilla reniformis* luciferase activity and to the DMSO control.

### 4.10. Exosome Isolation

Exosome isolation was performed as previously described [55]. Briefly, cells were washed with PBS three times and starved in serum-free medium for 24 h. The cell debris was removed from the cell culture media by centrifugation at 2000× *g* for 30 min. Then, cell media were centrifuged at 19,000× *g* for 30 min. After filtration of the supernatants using a filter of 0.2–0.8 µm pore size, the supernatant was centrifuged at 100,000× *g* for 70 min. Pellets were dissolved in PBS and centrifuged again at 100,000× *g* for 70 min. The isolated exosomes were subjected to Western blot analysis using antibodies against CD9 and CD63.

### 4.11. Statistical Analysis

Statistical analysis was carried out using the software package SPSS21 (SPSS, Chicago, IL, USA). Two-tailed chi-squared (χ2) and Fisher’s exact tests were conducted for evaluation of the association between protein expression and clinicopathological data. Student’s *t*-test was applied to analyze the differences between PD-L1 transfectants and mock control cells. Pearson correlation analysis was performed to evaluate the correlation between PD1 and PD-L1 protein expression in primary tumor samples. The *p*-values presented are two-sided. A *p*-value below 0.05 was defined as statistically significant.

## Figures and Tables

**Table 1:** Correlation between PD1 expression and clinicopathological parameters in primary lung tumor.

		PD1		*p*-Value	
		0–1	2–3		
Type	SCC	75	22	**0.0446**	Fisher’s
	ADC	22	1		
Gender	Male	93	22	0.735	Fisher’s
	Female	14	2		
pT	1–2	88	19	0.772	
	3–4	19	5		
pN	0	56	12	0.836	
	1–3	51	12		
Grade	1–2	71	13	0.261	
	3–4	36	11		
Survival time	<60	93	21	1.00	Fisher’s
	≥60	14	3		

Fisher’s: Fisher’s exact tests.

**Table 2:** Correlation between PD1 protein expression and tumor grading in lung SCC.

Type			PD1		*p*-Value
			0–1	2–3	
SCC	Grade	1–2	60	12	**0.016**
		3–4	12	10	

**Table 3:** Correlation between PD-L1 expression and clinicopathological parameters in primary lung tumor.

		PD-L1_TPS		*p*-Value	
		<1%	≥1%		
Type	SCC	67	28	**0.036**	Fisher’s
	ADC	22	2		
Gender	Male	83	31	0.56	Fisher’s
	Female	13	3		
pT	1–2	82	24	0.055	
	3–4	14	10		
pN	0	51	17	0.754	
	1–3	45	17		
Grade	1–2	60	22	0.819	
	3–4	36	12		
Survival time	<60	83	30	1.00	Fisher’s
	≥60	13	4		

TPS: Tumor Proportion Score; Fisher’s: Fisher’s exact tests.

**Table 4:** Pearson correlation coefficients between PD-L1 and PD1 protein expression in primary lung tumor.

	PD-L1 vs. PD1	
	NCSLC	SCC
Sample number	109	79
Correlation coefficient (r value)	0.400	0.465
Sig. (2-tailed) *p*	**0.000**	**0.000**

## Data Availability

Data is contained within the article or Supplementary Materials.

## Supplementary Materials

The supporting information can be downloaded at: www.mdpi.com/article/10.3390/ijms231911031/s1.

## Abbreviations

NSCLC	Non-small cell lung cancer
SCLC	Small cell lung cancer
ADC	Adenocarcinoma
SCC	Squamous cell carcinoma
PD1	Programmed cell death protein 1
PD-L1	Programmed cell death 1 ligand 1
5-Aza	5-aza-2′-deoxycytidine
TSA	Trichostatin A
HBEC	Human bronchial epithelial cells
miRNA	microRNA
TMA	Tissue microarray
TPS	Tumor proportion score (TPS)
siRNA	RNA interference
MAPK	Mitogen-activated protein kinase
LEF	Lymphoid enhancer binding factor
Sp5	Sp5 transcription factor
MMP14	Matrix metallopeptidase 14
CX43	Connexin 43
