# Supplementary figures and images for: Tumor-Intrinsic PD-L1 Exerts an Oncogenic Function through the Activation of the Wnt/β-Catenin Pathway in Human Non-Small Cell Lung Cancer
Source: Int J Mol Sci. 2022 Sep 20;23(19):11031. doi: 10.3390/ijms231911031 (PMC9569632; doi:10.3390/ijms231911031)

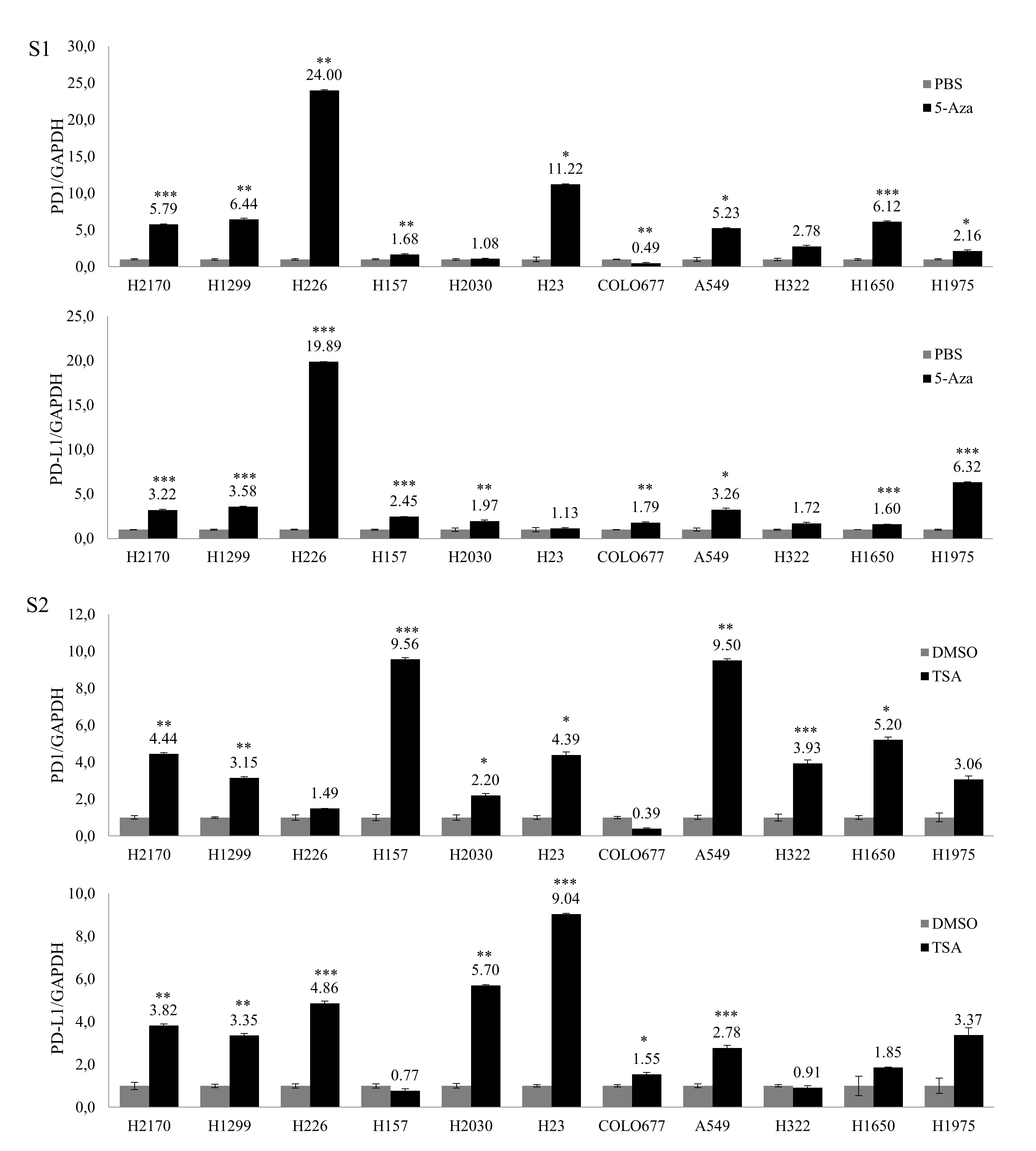

Supplement: Supplementary file 1 [file ijms-23-11031-s001.zip › Supplementary data S1-2.tif]

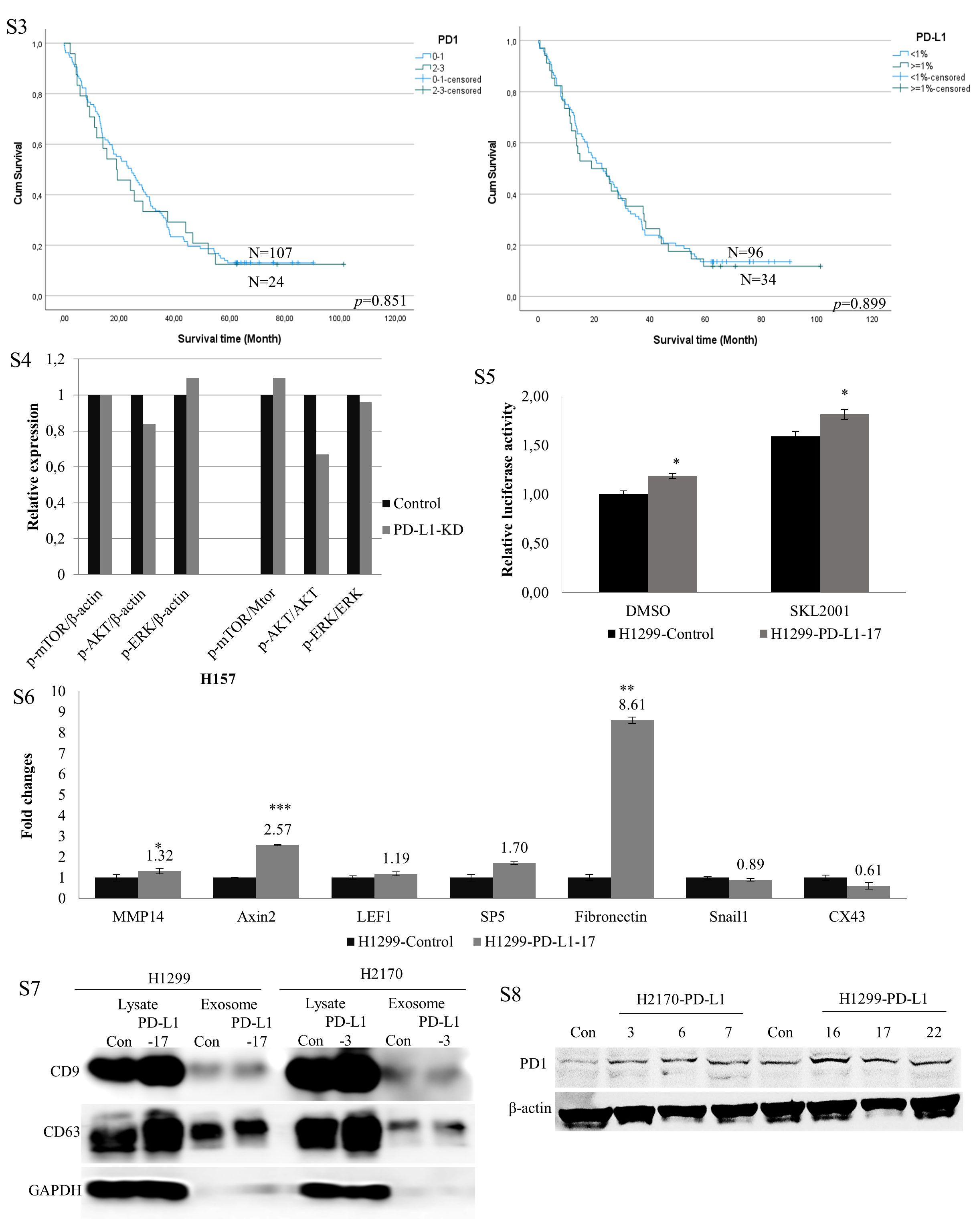

Supplement: Supplementary file 1 [file ijms-23-11031-s001.zip › Supplementary data S3-8.tif]
